# Heart Failure Syndromes: Different Definitions of Different Diseases—Do We Need Separate Guidelines? A Narrative Review

**DOI:** 10.3390/jcm14145090

**Published:** 2025-07-17

**Authors:** Massimo Romanò

**Affiliations:** 1 Columbus Clinic Center, Via Buonarroti 48, 20145 Milan, Italy; max.romano51@gmail.com; Tel.: +39-3488131566; 2 Organizing Committee Postgraduate Master in Palliative Care, University of Milan, 20122 Milan, Italy

**Keywords:** heart failure, heart failure classification, heart failure with reduced ejection fraction, heart failure with preserved ejection fraction, heart failure with mildly reduced ejection fraction, heart failure with normal ejection fraction

## Abstract

Heart failure (HF) is a well-known leading cause of mortality, associated with a high symptom burden in advanced stages, frequent hospitalizations, and increasing economic costs. HF is typically classified into three main subgroups, based on left ventricular ejection fraction (LVEF): HF with reduced ejection fraction (HFrEF), HF with mildly reduced ejection fraction (HFmrEF), and HF with preserved ejection fraction (HFpEF). Recently, two additional subgroups have been proposed: HF with improved ejection fraction (HFimpEF) and HF with supernormal ejection fraction (HFsnEF). These five phenotypes exhibit distinct risk factors, clinical presentations, therapeutic responses, and prognosis. However, the LVEF thresholds used to define these subgroups remain a subject of considerable debate, with significant differences in opinions among leading experts. A major criticism concerns the reliability of LVEF in accurately classifying HF subgroups. Due to substantial intra and interobserver variability, determining the appropriate therapy and prognosis can be challenging, particularly in patients with HFmrEF. Additionally, patients classified under HFpEF are often too heterogeneous to be effectively managed as a single group. This narrative review explores these issues, and suggests a possible need for a new approach to HF classification, one that involves revising the LVEF reference values for HF phenotypes and highlighting LVEF trajectories rather than relying on a single measurement. Moreover, in light of the relatively limited therapeutic options for patients with LVEF > 40%, a new, simplified classification may be proposed: HF with reduced EF (LVEF ≤ 40%), HF with below-normal EF (41% ≤ LVEF ≤ 55%), and HF with normal EF (LVEF > 55%). This mindset would better equip clinical cardiologists to manage the diverse spectrum of HF syndromes, always with the patient at the center.

## 1. Introduction

Heart failure (HF) is a leading cause of mortality, characterized by a high symptom burden in advanced stages, frequent hospitalizations, and rising economic costs [1].

The global median prevalence of HF in Europe is approximately 17 per 1000 individuals, while in the United States, the American Heart Association (AHA) estimated a prevalence of 2.5% in 2021 [2].

The median annual incidence is reported to be 3.2 per 1000 individuals per year [2].

HF is currently classified into three main phenotypes, based on left ventricular ejection fraction (LVEF) values, usually measured by two-dimensional transthoracic echocardiography (2D-TTE): (1) HF with reduced LVEF (HFrEF): (LVEF ≤ 40%), (2) HF with mildly reduced LVEF (HFmrEF): (LVEF 41–49%), and (3) HF with preserved LVEF (HFpEF): (LVEF ≥ 50%) [3,4]. A fourth phenotype, recognized as a distinct clinical entity, is HF with improved ejection fraction (HFimpEF), defined as prior LVEF ≤ 40% that has subsequently increased to ≥40% [5,6].

More recently, diagnostic criteria for HFimpEF have been redefined to include the following: (1) documented baseline LVEF < 40%; (2) an absolute improvement in LVEF ≥ 10%; and (3) a follow-up LVEF measurement > 40% [7].

These four phenotypes exhibit considerable heterogeneity, regarding epidemiology, clinical characteristics, prognosis, and therapeutic response.

Recently, some authors have challenged this classification and proposed alternative models [8,9,10,11,12,13,14].

The main criticism targets the narrow LVEF range defining HFmrEF [8,9], which is particularly sensitive to inter and intraobserver variability up to 7% [10], potentially resulting in a patient reclassification, between HFmrEF, HFrEF, or HFpEF, with significant clinical implications.

Moreover, the cutoff value of 40% for HFrEF was considered arbitrary by some, as major clinical trials have commonly used ≤35% as the threshold for reduced LVEF [8].

The concept of HFpEF has also been questioned, due to the heterogeneity of its clinical phenotypes, with some experts suggesting it be eliminated, in favor of HF with normal EF (HFnEF), defined as LVEF ≥ 60% [8].

Alternatively, it has been proposed that HFpEF be replaced by a combination of HFmrEF and HFnEF, with the latter defined as LVEF ≥ 55% in men and ≥60% in women [11].

Furthermore, a new phenotype, HF with supernormal LVEF (HFsnEF), defined as LVEF ≥ 65–70%, has been proposed, as patients in this category may have mortality rates comparable to those with LVEF ≤ 35–40% [11,12].

Emerging evidence also indicates an increased risk of all-cause mortality or HF hospitalization in patients with higher preserved LVEF (≥60%) [12,13,14], suggesting a U-shaped relationship between LVEF and mortality.

These inconsistencies in clearly defining HF phenotypes could have important clinical and prognostic implications.

This narrative review aims to discuss all these issues.

## 2. Limitations of LVEF-Based Classification

The limitations of the current HF classification system are well analyzed in a large Sweden Registry study by Christersson et al. [10], which highlights diagnostic uncertainty introduced by variability in LVEF measurements.

However, the criticism of using LVEF as a fundamental parameter for classifying different HF phenotypes [8,9] is counterbalanced by a strong defense of its utility by other authors [15].

Another important issue concerns the varying disease trajectories, particularly in patients with HFrEF, where pharmacological and device-based therapies can significantly alter clinical outcome [16].

A recent Clinical Consensus Statement from the Heart Failure Association (HFA) of the ESC, the Heart Failure Society of America (HFSA), and the Japanese Heart Failure Society (JHFS) adds further perspective to this complex topic [17].

The critical observations regarding the rigid definition of HF [8,9,10,11] are widely analyzed, debated, and largely accepted in this document.

Significant changes have been introduced concerning the definition of HF, including revised LVEF thresholds and a shift toward using LVEF trajectories rather than a single, baseline LVEF measurement, as a more meaningful clinical parameter, in line with the Japanese Guidelines for the Diagnosis and Treatment of HF [18].

Consequently three new HF types have been proposed: HF with unchanged LVEF, HF with worsening LVEF, and HF with recovered LVEF.

**Figure 1:**
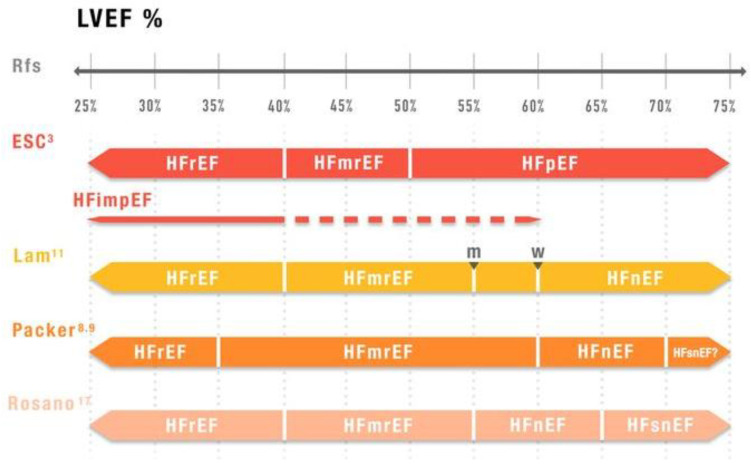
**Different HF classification based on LVEF values in main studies**. LVEF: left ventricular ejection fraction; HFrEF: heart failure with reduced ejection fraction; HFmEF: heart failure with mildly reduced ejection fraction; HFpEF: heart failure with preserved ejection fraction; HFimpEF: heart failure with improved ejection fraction; HFnEF: heart failure with normal ejection fraction; HFsnEF: heart failure with supernormal ejection fraction; m: males; w: women; Rfs: references [3,8,9,11,17].

These alternative classification approaches are illustrated in Figure 1.

Therefore, considerable uncertainty exists in this field, potentially causing confusion among cardiologists.

HF seems to have multiple forms, with each following different disease trajectories [16], raising the question of whether HF guidelines should aim for a single all-inclusive model or adopt a more differentiated, phenotype-specific approach.

The epidemiology of HF phenotypes varies across studies, primarily due to the incomplete availability of LVEF data and differing HF classifications based on LVEF values, as reported in observational studies and registries [19,20].

An example illustrating the importance of this variability is a report by Savarese et al. [21], in which applying the 2021 ESC Guidelines classification, compared to the 2016 version, resulted in 7% of patients with an LVEF = 40% being reclassified from HFmrEF to HFrEF.

In this regard, it is important to highlight the limitations inherent to the data collection process of the registries, which could result in the loss of generalizability and introduce bias, leading to erroneous or invalid results.

Table 1 presents key data from various registries and studies [14,19,20,22,23,24,25,26,27].

However, the data in Table 1 may not reflect recent trends, as the prevalence of HFpEF seems to be increasing, reaching up to approximately 50%, due to increasing age, obesity, and other associated comorbidities [28].

While the LVEF cutoff are consistent across most references (HFrEF: EF < 40%, HFmrEF: LVEF 40–49%, HFpEF: LVEF ≥ 50), the ESC-HF Long-Term Registry [19] uses slightly different definitions: HFmrEF as LVEF 40–50% and HFpEF as LVEF > 50%.

The table highlights the significant wide epidemiological variability and the differing LVEF values used across studies.

These findings emphasize how small differences in LVEF resulting from varying classification criteria, different classification, or inter- and intraobserver variability can influence therapeutic decisions and patient communication, particularly regarding therapies and prognosis, which differ across HF phenotypes.

## 3. Phenotype-Specific Evidence and Challenges

1. ***HFrEF***

The ESC and AHA/ACC/HFSA Guidelines define HFrEF as a condition with an LVEF ≤ 40% [3,4].

The origins of this threshold could stem from historical randomized clinical trials (RCTs):

1. The Multicenter Postinfarction Research Group analyzed 886 patients post myocardial infarction (MI) and identified LVEF < 40% as one of the four independent risk factors for mortality [29].
2. The Survival And Ventricular Enlargement (SAVE) trial demonstrated significant reductions in total and cardiovascular mortality and morbidity in patients with recent MI and LVEF ≤ 40% who were treated with captopril [30]. In this trial, the 40% cutoff was based on earlier studies showing increased mortality and sudden cardiac death in patients with LVEF ≤ 40% [31].

However, these trials primarily include patients with recent MI and HF, mainly males, and do not represent the broader population of individuals with cardiac failure today.

The historical trials that enrolled patients with HF regardless of etiology generally used a lower LVEF threshold (≤35%) to evaluate the efficacy of implantable cardioverter defibrillators (ICDs) [32], cardiac resynchronization therapy (CRT) [32], or pharmacological treatments [3,4] in reducing overall mortality (Table 2 and Table 3).

Recent trials involving sodium–glucose cotransporter 2 inhibitors (SGLT2i) used a cutoff of LVEF ≤ 40%, while the Victoria study with vericiguat applied a cutoff of <45%, despite targeting patients with HFrEF [33].

This choice appears inconsistent and lacks clear justification.

Further confusion arises from the Paradigm-HF trial (prospective comparison of angiotensin receptor neprilysin inhibitors [ARNI] with angiotensin converting enzyme inhibitors [ACEI] to determine the impact on global mortality and morbidity in HF) [34]. Initially the trial included patients with LVEF ≤ 40% but the protocol was subsequently amended, redefining HFrEF as LVEF ≤ 35% [4].

The benefits, particularly in reducing total and cardiovascular mortality, were more evident in patients with LVEF ≤ 35%.

Table 2 and Table 3 show that the mean LVEF of patients enrolled in both the drug or device trials was significantly below 35%.

Despite these concerns, current guidelines recommend the four pillars (betablockers, mineralocorticoid receptor antagonists [MRA], ACE-I/angiotensin receptor blockers [ARB], ARNI), while in selected patients, CRT, ICD, or CRTD implantation is also advised in patients with HFrEF, defined as LVEF ≤ 40 [3,4]. All these treatment options reduce mortality.

Moreover, it should be remembered that cardiac implantable electronic devices are a cornerstone of HFrEF therapy, even with the abovementioned limits of LVEF measurement. The correct indication will be improved with the wider use of other imaging techniques, as described below.

More recently, new drugs have been tested in HFrEF patients, such as vericiguat, a soluble guanylate cyclase stimulator [33], and omecamtiv mecarbil, a cardiac myosin activator [35].

In both studies, patients who received the drugs showed a lower incidence of a composite of heart failure hospitalizations or death from cardiovascular causes than among those who received placebo.

The efficacy of emerging therapies was analyzed in a recent systematic review and network meta-analysis, with particular attention to specific subgroups of patients: elderly (>65 years and >75 years), patients with chronic kidney disease, diabetes mellitus, coronary artery disease, NYHA class III/IV, women, or patients already treated or not treated with sacubitril/valsartan [36].

The primary end-point analyzed was a composite of cardiovascular death and HF hospitalization, significantly reduced in some subgroups. The relative risk reductions of each drug in each subgroup are shown in Table 4.

2. ***HFmrEF***

HFmrEF is classified with an LVEF of 41–49% [3,4].

This specific phenotype has an interesting history. It was first considered in the ACCF/AHA 2013 HF guidelines, where LVEF values of 40–50% were included under the broader phenotype of HFpEF [37]. However, many patients within this range exhibited clinical characteristics more similar to HFrEF than to HFpEF. Consequently, the initial definition of HF with mid-range LVEF was later renamed as HF with mildly reduced EF [3].

Moreover, several intriguing aspects exist [38]:

- HFmrEF may be defined as an “HF gray zone” [39], due to its heterogeneity. These patients tend to have a similar prevalence of certain comorbidities (e.g., ischemic heart disease) as seen in HFrEF but lower rates of others (e.g., older age, arterial hypertension, atrial fibrillation, chronic kidney disease) compared to HFpEF.
- Cardiovascular outcomes are generally better than in HFrEF, but the risk of non-cardiovascular adverse events is higher than in HFrEF and comparable to HFpEF [38].
- Swedish/Dutch registries identified six distinct clusters among patients with HFmrEF, each with different pathophysiology, clinical patterns, therapeutic responses and prognosis [40] (Table 5).
- RCTs exclusively targeting patients with HFmrEF are lacking. Most studies have focused on patients with HFmrEF/HFpEF, therefore with LVEF > 40%.
- Recent focused update of ESC HF Guidelines [3] recommend SGLT2i with class 1A indication, while the ACC/AHA/HFSA Guidelines assign a class 2a/B-R rating [4]. These agents have demonstrated a significant reduction in composite end-points of cardiovascular death or HF hospitalization in RCTs. However, the benefits were primarily driven by reduced HF hospitalizations. Regarding other drug classes, the HF Guidelines provide a class 2b, level C recommendation for ACEI/ARB/ARNI, MRA, and betablockers [3,4].
- More recently the FINEARTS-HF trial demonstrated that the nonsteroidal MRA finerenone significantly reduced worsening HF or cardiovascular death in patients with HFmrEF/HFpEF, with the benefits fully attributable to reduced worsening HF [41].

An isolated report from the Swedish HF Registry suggested a potential benefit of renin–angiotensin system inhibitors/ARNI and betablockers in reducing mortality and morbidity. However, the findings have some statistical limitations [42].

Therefore, HFmrEF seems to be a particular clinical entity, with a narrow LVEF values interval, so patients with HFmrEF can easily shift into the HFrEF or HFpEF categories, not due to true changes in cardiac function, but due to inter- or intraobserver variability in LVEF measurements. This variability, with a 75–80% likelihood of non-reproducibility [10], strongly impacts the accurate implementation of guideline-recommended treatments.

Finally, even the most recent trials investigating semaglutide in HF have pooled patients with HFmrEF and HFpEF [43]. Despite being grouped together, these two phenotypes differ significantly in pathophysiology and clinical presentation.

Therefore, this merger appears to be inconsistent.

3. ***HFpEF***

This HF class is present in up to 50% of patients hospitalized for HF and is even more complex to analyze than the previous two categories.

Briefly, it encompasses many subphenotypes [44], primary and secondary, each with distinct physiopathological complexity and clinical profiles, but still lacks a demonstrated effective therapy for reducing mortality. SGLT2i [3,4], finrenone [41], and semaglutide [43] have shown benefits only in reducing HF hospitalizations or worsening HF and in improving quality of life [43].

According to a recent review, the most frequently reported phenotypes include three [45]: an older (vascular ageing phenotype), a metabolic–obese phenotype, and a relatively younger (low BNP phenotype).

The first phenotype is commonly characterized by arterial hypertension, and often coexists with chronic kidney disease. These comorbidities are also present in the second phenotype, where type 2 diabetes predominates. Systemic inflammation underlies both phenotypes.

The third phenotype, the younger, low BNP group, shares some clinical features, such as obesity, and may benefit from the use of an MRA, such as spironolactone [45].

Prognosis is poorest in the first phenotype and better in the third, and intermediate in the second.

In the Swedish/Dutch HF Registries, five distinct phenotypic clusters have been identified [46].

In a clinical cohort of patients hospitalized with HFpEF, rates of cardiovascular and non-cardiovascular deaths and hospitalizations were found to be comparable.

Cardiovascular deaths were primarily predicted by HF severity, while non-cardiovascular deaths were primarily linked to anemia and prior stroke [47].

This heterogeneity necessitates multiple pharmacological treatments, which often have reciprocal contraindications, making it clear why no single therapy has been shown to reduce mortality effectively.

Some intriguing pathophysiological correlations have been proposed in some phenotypes: (1) non-alcoholic steatohepatitis (NASH) of the heart [48], linked to the cardiometabolic HFpEF phenotype, characterized by lipid accumulation, systemic inflammation, progressive fibrosis, and organ dysfunction, mirroring the pathogenesis to NASH or (2) dementia of the heart [49], associated with the ageing phenotype and its multiple comorbidities, leading to consequent organ dysfunction similar to dementia, mainly driven by vascular damage.

Additionally, significant differences exist in clinical characteristics within this phenotype, depending on whether LVEF is defined as normal (≥55% in men or ≥60% in women) [11] or preserved (LVEF ≥ 50%) [50].

Conclusions from the major trials on HFpEF treatment show a significant reduction in composite end-point of death or hospitalizations/worsening HF. However, no drug or devices has demonstrated capacity to reduce mortality.

Moreover the therapeutic effects of ARNI and SGLT2i appear reduced in patients with LVEF of 55–60% [51].

Currently, the treatment goal in patients with HFpEF is to manage comorbidities, reduce hospitalizations, and improve quality of life [51].

Ultimately, HFpEF remains a puzzle, composed of different disease entities, raising an important question: do we need separate guidelines for each phenotype?

4. ***HFimpEF***

The incidence of HFimpEF is approximately 20% in the largest studies [27,52], although rates as high as 60% have been reported [53].

In the MECKI registry, the principal characteristics of this group include higher prevalence of non-ischemic etiology, female sex, and elevated blood pressure levels [52].

The prognosis of these patients is better than that of those with persistently reduced LVEF [51], but the risk of adverse events remains significantly high, with cardiovascular mortality rates of 26.6 per 1000 person-years [53].

Patients with HFimpEF are treated with guideline-directed medical therapy (GDMT), with special attention given to SGLT2i [27], and also with ICDs.

However, these patients are largely heterogeneous, with different underlying etiology and prognosis.

The HF relapse is widely variable, according to the different trials: the follow-up duration ranges from 16 months to 7 years [53].

The incidence of relapse ranges from 13.8% at 1 year to 80% at 7 years.

There is discussion in the literature of how long to continue with GDMT, and also of the eventuality of withdrawing the ICD therapy, in highly selected patients [53].

Patients with LVEF ≥ 50%, without clinical signs or symptoms of HF, normal imaging parameters (e.g., CMRI), normal body-mass-index-adjusted NT-proBNP, normal ECG findings, and reversible etiology, are considered at low risk, and may be evaluated for partial de-escalating GDMT [53].

5. ***HFsnHF***

This particular heterogenous HF phenotype (LVEF ≥ 65%) gained attention some years ago when a U-shaped relationship between EF and mortality was proposed, suggesting a higher risk in patients with LVEF > 60–65% [14].

The prevalence of this phenotype ranges between 12% and 17% [14].

In a recent Swedish registry study (which included patients with LVEF ≥ 60%), this relationship was confirmed only in unadjusted analysis, showing increased rates of hospitalizations. However, after adjusting for confounders, no significant association was found [54].

Furthermore, it is interesting to note that having HFsnEF does not mean to have normal systolic function: in these patients, there is an abnormal left ventricular stiffening, coupled with arterial stiffening, leading to a significant increased afterload and myocardial oxygen consumption [13].

Moreover, as with other patients with HFpEF, common confounders were hypertension, female sex, valvular disease, older age, and hypertrophic cardiomyopathy.

## 4. Emerging Tools to Improve Phenotyping Hf Patients

Other emerging tools may overcome the limits of 2D-TTE in accurately defining the HF phenotypes. They are briefly analyzed.

a. Three-dimensional speckle-tracking echocardiography allows for a better evaluation of global and regional function than 2D-TTE, particularly in patients with HF [55].
b. Cardiac magnetic resonance imaging (CMRI) is considered the gold standard for the assessment of cardiac volumes and LVEF, for tissue characterization and mechanical dyssynchrony in patients with HFrEF [56]. This is essential information in order to provide a correct indication regarding device implantation.

In the general population, CMRI defines cardiac volumes and function more accurately than 2D-TTE, with lower interobserver variability [57]. CMRI LVEF is 10% higher than 2D-TTE, with significant clinical consequences, regarding drugs and device implementation, and different prognoses [56,57].

However, the costs and limited availability do not allow for a wider use of CMRI.

c. In selected patients with coronary artery disease, nuclear imaging techniques, such as equilibrium blood pool ventriculography or gated single-photon emission CT (SPECT), may be useful, particularly when contemporary information on myocardial perfusion is needed [56].
d. Artificial intelligence (AI) and machine learning is transforming the cardiovascular imaging, dramatically improving the image acquisition process. AI may be useful in echocardiography, CMI, cardiac and coronary computer tomography, and nuclear cardiology [58].

Unsupervised ML algorithms are preferred over supervised ML, because they utilize unlabeled data. The application of the unsupervised ML models in HF may contribute to better identify clusters of phenotypes, especially in HFpEF.

AI-based echocardiography may also reduce the interobserver variability, improving the correct diagnosis of HF [58].

e. Moreover, omics technologies may further elucidate the underlying genetic mechanisms of HF.

The omics data can incorporate genomics, transcriptomics, epigenomics, proteomics, and metabolomics.

Omics research could provide the basis for studies potentially linked with HF development and progression, and possible therapeutic implications.

Transcriptomic analysis provides information about all the RNA transcripts of an organism, offering the possibility of measuring gene expression in different HF phenotypes (HFpEF vs. HFrEF). Novel biomarkers could be identified and contribute to a better definition of HF subtypes [59].

Moreover, the application of AI to multiomics technologies would be an opportunity to improve a personalized therapeutic approach.

## 5. Alternative Classifications and Future Directions

Recently, many authors have questioned the traditional classification of HF based solely on LVEF, as LVEF alone no longer provides a comprehensive assessment of the syndrome [8,9,10,11,12,13,14,17].

The shift is driven by multiple factors, including the evident clinical heterogeneity among patients, particularly those with HFmrEF and HFpEF [40,44], and distinct pathophysiologies underlying these two phenotypes [8,9].

These differences potentially contribute to the limited efficacy of drug therapies in reducing mortality as well as the increasing recognition of comorbidities as major determinants of clinical course and prognosis.

Moreover, additional limitations of using LVEF as a sole criterion include the narrow LVEF definition of HFmrEF, the significant inter and intraobserver variability in LVEF measurement obtained by 2D-TTE, and the load and rhythm dependence of LVEF [9,10,21].

In contrast, Lund et al. [15] have recently reaffirmed the utility of echocardiographic LVEF and the current three-phenotype classification system.

However, an increasing number of papers are exploring alternative approaches to HF classification, including redefining LVEF cutoffs [8,9,10,11,17] or proposing etiologic classifications, although these tend to be more complex and less practical for widespread clinical use [60].

Another critical point in reclassifying chronic HF is the variations in the efficacy of drugs and devices across different HF phenotypes.

While the so-called “four pillars” of HFrEF therapy, along with ICD and CRT, have been shown to reduce mortality in patients with HFrEF, no treatment has demonstrated mortality benefit in HFmrEF or HFpEF. Rather, in these group, the benefits have been limited to reductions in worsening HF or hospitalizations.

The extension of ARNI, and especially SGLT2i and MRA, across the full spectrum of HF is primary based on their ability to reduce composite end-points of death, worsening HF, or HF hospitalization [11,61].

Docherty et al. [61] analyzed the effects of the “four pillars” across all patients with HF, regardless of LVEF.

However, they emphasized that these results were “derived from post-hoc analyses and should be considered as hypothesis-generating” [61].

Reducing hospitalizations and/or episodes of worsening HF remains a crucial therapeutic goal in HF treatment, regardless of LVEF, due to their strong association with mortality. Still, efforts to identify therapies that consistently reduce mortality across the entire HF spectrum have so far been disappointing.

The recent Clinical Consensus Statement from the HFA of the ESC, HFSA, and JHFS [17] proposes a new classification of HF (Figure 1).

This new framework moves beyond rigid LVEF cutoffs, reclassifying HFmrEF into broader LVEF values (40–55%) and introducing a phenotype of HF with normal LVEF (HFnEF).

It also introduces, for the first time, a distinct phenotype of supernormal LVEF (HFsnEF).

This phenotype deserves a clinical recognition, as it is associated with a high burden of comorbidities and poorer survival [14], particularly in the presence of genetic predisposition [62].

Moreover, it does not seem to benefit from treatment with SGLT2i [63].

There is increasing interest and research to provide a phenotype-based therapy for patients with HFpEF, who represent approximately 50% of HF patients.

Fluid retention, low LVEF values, and/or metabolic disorders, such as obesity or type 2 diabetes mellitus, could lead to more efficacious medical therapy [64].

Patients with HFmrEF remain a Cinderella in the field of HF, due to a lack of consensus in classification and different phenotypes, some quite similar to HFrEF, others similar to HFpEF.

A possible and desirable overcoming of these gaps could lead to a more tailored treatment.

## 6. Conclusions

Many questions arise from the analysis of the data presented in this review.

The first concerns the wide variability in LVEF cutoffs proposed by different authors, as depicted in Figure 1.

This variability raises doubts and perhaps a little degree of confusion among cardiologists.

How should a patient with an LVEF of 55% be defined: HFmrEF [8,9,11,17], HFpEF [3], or HFnEF [17]?

Is it merely a terminological issue, with limited clinical or therapeutical implications or could it prompt a fundamental reassessment of our beliefs?

If a patient initially presents with an LVEF of 40%, how much time should elapse before re-evaluation and definition of clinical trajectory: 3, 6, 12 months, and how should this influence reclassification [17]?

Furthermore, how can we design future drug/device trials without a universally accepted HF classification?

Should we resign ourselves, at least for the foreseeable future, to the notion that no new treatment will significantly reduce mortality in patients with LVEF ≤ 40%?

This last question seems a natural consequence of the current difficulty in identifying clean therapeutic targets within the highly heterogeneous population of patients with LVEF >40%.

**Figure 2:**
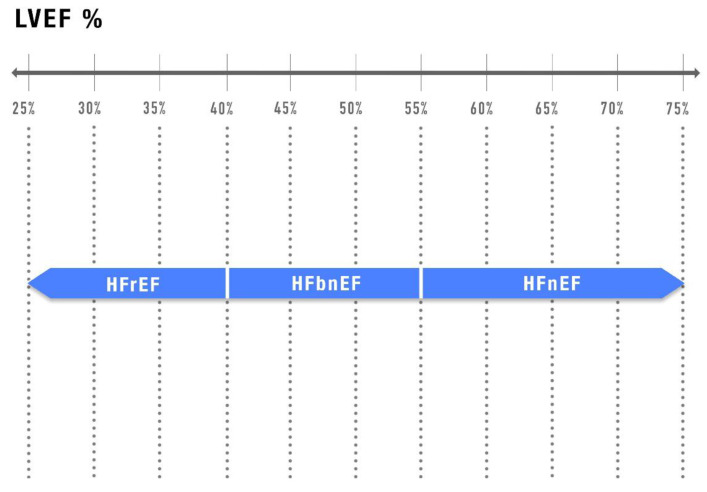
**New proposed HF classification.** HFrEF: Heart failure with reduced ejection fraction; HFbnEF: heart failure with below normal ejection fraction; HFnEF: heart failure with normal ejection fraction.

Perhaps a simplified reclassification of HF could be proposed (Figure 2): HF with reduced EF (LVEF ≤ 40%), HF with below-normal EF (41% ≤ LVEF ≤ 55%) [61], and HF with normal EF (LVEF > 55%), even if it involves some limitations in the use of “normal” LVEF, defined by the American Society of Echocardiography and European Society of Echocardiography, based on data from the general population [65].

Finally, given that we are dealing with multiple classifications of distinct disease entities, it may be time to develop new, differentiated HF guidelines, ones that adopt a more translational and personalized approach, as recently proposed in an HFA of ESC statement on HF and obesity [66].

Such a mindset could help clinical cardiologists in navigating the huge complexity of HF syndromes.

## Figures and Tables

**Table 1:** Prevalence of different phenotypes of heart failure. The sources refer to the main trials that analyzed the prevalence of the distinct heart failure phenotypes. In brackets there are the numbers of patients enrolled in each trial.

Sources (pts)	HFrEF	HFmEF	HFpEF	HFimpEF	HFsnEF
ESC-HF-LT [19] (20896)	62%	14%	24%		
Swedish HF Registry [20] (76453)	53%	23%	24%		
OPTIMIZE-HF Registry [22] (41217)	49%	17%	24%		
GWTG-HF study [23] (99825)	49%	13%	38%		
G-CHF [24] (23047)	54%	21%	24%		
Management of Cardiac Failure program in Northern Sydney Australia (5236) [25]	47.8%	14.9%	37.4%		
Deliver Trial (1151) [27]				18%	
Wehner (203135) [14]					12%

**Table 2:** Left ventricular ejection fraction (LVEF) values in main randomized clinical trials on devices in patients with left ventricular dysfunction.

Trials	Device	LVEF (%)
MADIT II	ICD	≤30
SCD-HeFT	ICD	≤35 (median 25)
CARE HF	CRT	≤35 (median 25)

ICD: Implantable cardioverter defibrillator. CRT: Cardiac resynchronization therapy. From reference [32].

**Table 3:** Left ventricular ejection fraction (LVEF) cutoffs in main randomized clinical trials on drugs in heart failure.

Trials	Drug	LVEF (%)
MERIT HF	Metoprolol XL	≤40 (mean 28 ± 7)
CIBIS II	Bisoprolol	≤35 (mean 27 ± 6)
COPERNICUS	Carvedilol	<25 (mean 19.8 ± 4)
CONSENSUS	Enalapril	NYHA IV
SOLVD	Enalapril	≤35 (mean 24.9)
Val-HeFT	Valsartan	<40 (mean 26.6 ± 7.3)
RALES	Spironolactone	≤35 (mean 25.2 ± 6.8)
EMPHASIS-HF	Eplerenone	≤35 (mean 26.2 ± 4.6)
PARADIGM-HF	Sacubitril/Valsartan	≤40, amended to 35 (mean 29.6 ± 6.1)
DAPA-HF	Dapaglifozin	≤40 (mean 31.2 ± 6.7)
EMPEROR-Reduced	Empaglifozin	≤40 (mean 31.2 ± 6.7)
VICTORIA	Vericiguat	<45 (86% < 40)
GALACTIC-HF	Omecamtiv mecarbil	≤35 (mean 26.6 ± 6.3)

The data in brackets refer to mean LVEF, except Consensus and Victoria trials. From references [3,4].

**Table 4:** Relative risk reduction of new drugs in a specific subgroup of patients with HFrEF.

Drug	CKD	CAD	Age > 65 Y	Age > 75 Y	NYHA III/IV	DM	Already ARNI	No ARNI	Women
Dapaglifozin	**0.72**	**0.77**	**0.72**	**0.68**	0.90	**0.75**	0.75	**0.74**	0.79
Empaglifozin	0.83	**0.82**	**0.78**	0.86	0.83	**0.72**	**0.64**	**0.77**	**0.59**
ARNI	**0.79**	0.90	**0.80**	0.86					
Placebo	1.00	1.00	1.00	1.00	1.00	1.00	1.00	1.00	1.00
Omecamtiv	0.98	**0.90**	0.94		**0.88**	0.93	0.97	0.91	0.95
Vericiguat	**0.84**	0.92	0.94	1.04	**0.87**		0.88	**0.90**	0.88

The relative risk reductions of the new drugs in a specific subgroups of patients are shown in the boxes. In bold the statistically significant values are highlighted. CKD: Chronic kidney disease; CAD: coronary artery disease; DM: diabetes mellitus; ARNI angiotensin receptor-neprilysin inhibitor. Adapted from Lavalle et al. [36].

**Table 5:** Main characteristics of different clusters in patients with HFmrEF.

Cluster	Young–Low Comorbidity	Ischemic	Atrial Fibrillation	Wide QRS-Device	Metabolic	Cardio-Renal
%	17	13	20	9	19	22
NYHA class (% III/IV)	9.3	7.8	24.7	41.6	43.1	46.6
IHD (%)	37.7	82.9	21.7	70.1	75.2	64.7
AF (%)	26.5	15.3	87.4	85.7	61.3	74.7
Hypertension (%)	43.1	68.7	54.3	71.3	97.7	91.8
Diabetes mellitus (%)	14.0	10.0	3.8	26.8	84.1	22.7

IHD: Ischemic heart disease. AF: Atrial fibrillation. Adapted from Mejis et al. [40].

## References

[1] Savarese G., Becher P.M., Lund L.H., Seferovic P., Rosano G.M.C., Coats A.J.S. (2023). Global burden of heart failure: A comprehensive and updated review of epidemiology. Cardiovasc. Res..

[2] Seferovic P.M., Vardas P., Jankowska E.A., Maggioni A.P., Timmis A., Milinković I., Polovina M., Lainščak M., Timmis A., Huculeci R. (2021). The Heart Failure Association atlas: Heart failure epidemiology and management statistics 2019. Eur. J. Heart Fail..

[3] McDonagh T.A., Metra M., Adamo M., Gardner R.S., Baumbach A., Böhm M., Burri H., Butler J., Čelutkienė J., Chioncel O. (2023). 2021 ESC Guidelines for the diagnosis and treatment of acute and chronic heart failure: Developed by the Task Force for the diagnosis and treatment of acute and chronic heart failure of the European Society of Cardiology (ESC) with the special contribution of the Heart Failure Association (HFA) of the ESC. Eur. Heart J..

[4] Heidenreich P.A., Bozkurt B., Aguilar D., Allen L.A., Byun J.J., Colvin M.M., Deswal A., Drazner M.H., Dunlay S.M., Evers L.R. (2022). 2022 AHA/ACC/HFSA guideline for the management of heart failure: A report of the american college of Cardiology/american heart association joint committee on clinical practice guidelines. Circulation.

[5] Bozkurt B., Coats A.J., Tsutsui H., Abdelhamid M., Adamopoulos S., Albert N., Anker S.D., Atherton J., Böhm M., Butler J. (2021). Universal definition and classification of heart failure: A report of the heart failure society of america, heart failure association of the european society of cardiology, Japanese heart failure society and writing committee of the universal definition of heart failure. Eur. J. Heart Fail..

[6] Kalogeropoulos A.P., Fonarow G.C., Georgiopoulou V., Burkman G., Siwamogsatham S., Patel A., Papadimitriou L., Butler J. (2016). Characteristics and outcomes of adult outpatients with heart failure and improved or recovered ejection fraction. JAMA Cardiol..

[7] Wilcox J.E., Fang J.C., Margulies K.B., Mann D.L. (2020). Heart Failure with Recovered Left Ventricular Ejection Fraction: JACC Scientific Expert Panel. J. Am. Coll. Cardiol..

[8] Packer M. (2023). A reclassification of heart failure based on recognition of heart failure with normal to supernormal ejection fraction, a clinically common form of cardiac contracture, with distinctive pathophysiological and therapeutic features. Eur. J. Heart Fail..

[9] Packer M. (2024). Left Ventricular Ejection Fraction in Heart Failure: Crazy, Stupid Love-and Maybe, Redemption. J. Am. Heart Assoc..

[10] Christersson M., Gustafsson S., Lampa E., Almstedt M., Cars T., Bodegård J., Arefalk G., Sundström J. (2024). Usefulness of Heart Failure Categories Based on Left Ventricular Ejection Fraction. J. Am. Heart Assoc..

[11] Lam C.S.P., Solomon S.D. (2021). Classification of Heart Failure According to Ejection Fraction: JACC Review Topic of the Week. J. Am. Coll. Cardiol..

[12] Ohte N., Kikuchi S., Iwahashi N., Kinugasa Y., Dohi K., Takase H., Masai K., Inoue K., Okumura T., Hachiya K. (2023). Unfavourable outcomes in patients with heart failure with higher preserved left ventricular ejection fraction. Eur. Heart J. Cardiovasc. Imaging.

[13] Smiseth O.A., Fernandes J.F., Lamata P. (2023). The challenge of understanting heart failure with supernormal left ventricular ejection fraction: Time for building the patient’s digital twin. Eur. Heart J. Cardiovasc. Imaging.

[14] Wehner G.J., Jing L., Haggerty C.M., Suever J.D., Leader J.B., Hartzel D.N., Kirchner H.L., Manus J.N.A., James N., Ayar Z. (2020). Routinely reported ejection fraction and mortality in clinical practice: Where does the nadir of risk lie?. Eur. Heart J..

[15] Lund L.H., Pitt B., Metra M. (2022). Left ventricular ejection fraction as the primary heart failure phenotyping parameter. Eur. J. Heart Fail..

[16] Romanò M. (2024). New Disease Trajectories of Heart Failure: Challenges in Determining the Ideal Timing of Palliative Care Implementation. J. Palliat. Med..

[17] Rosano G.M.C., Teerlink J.R., Kinugawa K., Bayes-Genis A., Chioncel O., Fang J., Greenberg B., Ibrahim N.E., Imamura T., Inomata T. The use of Left Ventricular Ejection Fraction in the Diagnosis and Management of Heart Failure. A Clinical Consensus Statement of the Heart Failure Association (HFA) of the ESC, the Heart Failure Society of America (HFSA), and the Japanese Heart Failure Society (JHFS). Eur. J. Card. Fail..

[18] Tsutsui H., Ide T., Ito H., Kihara Y., Kinugawa K., Kinugawa S., Makaya M., Murohara T., Node K., Saito Y. (2021). JCS/JHFS 2021 Guideline Focused Update on Diagnosis and Treatment of Acute and Chronic Heart Failure. Circ. J..

[19] Chioncel O., Lainscak M., Seferovic P.M., Anker S.D., Crespo-Leiro M.G., Harjola V., Parissis J., Laroche C., Piepoli M.F., Fonseca C. (2017). Epidemiology and one-year outcomes in patients with chronic heart failure and preserved, mid-range and reduced ejection fraction: An analysis of the ESC Heart Failure Long-Term Registry. Eur. J. Heart Fail..

[20] Stolfo D., Lund L.H., Benson L., Hage C., Sinagra G., Dahlström U., Savarese G. (2022). Persistent high burden of heart failure across the ejection fraction spectrum in a nationwide setting. J. Am. Heart Assoc..

[21] Savarese G., Gatti P., Benson L., Adamo M., Chioncel O., Crespo-Leiro M.G., Anker S.D., Coats A.J.S., Filippatos G., Lainscak M. (2024). Left ventricular ejection fraction digit bias and reclassification of heart failure with mildly reduced vs reduced ejection fraction based on the 2021 definition and classification of heart failure. Am. Heart J..

[22] Fonarow G.C., Stough W.G., Abraham W.T., Albert N.M., Gheorghiade M., Greenberg B.H., O’Connor C.M., Sun J.L., Yancy C.W., Young J.B. (2007). Characteristics, treatments, and outcomes of patients with preserved systolic function hospitalized for heart failure: A report from the OPTIMIZE-HF registry. J. Am. Coll. Cardiol..

[23] Kapoor J.R., Kapoor R., Ju C., Eapen Z.J., Hernandez A.F., Butler J., Yancy C.W., Fonarow G.C. (2016). Precipitating clinical factors, heart failure characterization, and outcomes in patients hospitalized with heart failure with reduced, borderline, and preserved ejection fraction. JACC Heart Fail..

[24] Joseph P., Dokainish H., McCready T., Budaj A., Roy A., Ertl G., Gomez-Mesa J.E., Leong D., Ezekowitz J., Hage C. (2020). A multinational registry to study the characteristics and outcomes of heart failure patients: The global congestive heart failure (G-CHF) registry. Am. Heart J..

[25] Wang N., Hales S., Barin E., Tofler G. (2018). Characteristics and outcome for heart failure patients with mid-range ejection fraction. J. Cardiovasc. Med..

[26] Horiuchi Y., Asami M., Ide T., Yahagi K., Komiyama K., Yuzawa H., Tanaka J., Aoki J., Matsushima S., Tohyama T. (2023). Prevalence, characteristics and cardiovascular and non-cardiovascular outcomes in patients with heart failure with supra-normal ejection fraction: Insight from the JROADHF study. Eur. J. Heart Fail..

[27] Vardeny O., Desai A.S., Jhund P.S., Fang J.C., Claggett B., de Boer R.A., Fernandez A.F., Inzucchi S.E., Kosiborod M.N., Lam C.S.P. (2024). Dapagliflozin and Mode of Death in Heart Failure with Improved Ejection Fraction: A Post Hoc Analysis of the DELIVER Trial. JAMA Cardiol..

[28] Borlaug B.A., Sharma K., Shah S.J., Ho J.E. (2023). Heart Failure with Preserved Ejection Fraction: JACC Scientific Statement. J. Am. Coll. Cardiol..

[29] The Multicenter Postinfarction Research Group (1983). Risk Stratification and Survival after Myocardial Infarction. N. Engl. J. Med..

[30] Pfeffer M.A., Braunwald E., Moye L.A., Basta L., Brown E.J., Cuddy T.E., Davis B.R., Geltman E.M., Goldman S., Flaker G.C. (1992). Effect of captopril on mortality and morbidity in patients with left ventricular dysfunction after myocardial infarction: Results of the Survival and Ventricular Enlargement Trial. N. Engl. J. Med..

[31] Norris R.M., Barnaby P.F., Brandt P.W., Geary G.G., Whitlock R.M., Wild C.J., Barratt-Boyes B.G. (1984). Prognosis after recovery from first acute myocardial infarction: Determinants of reinfarction and sudden death. Am. J. Cardiol..

[32] Zeppenfeld K., Tfelt-Hansen J., de Riva M., Winkel B.G., Behr E.R., Blom N.A., Charron P., Corrado D., Dagres N., de Chillou C. (2022). ESC Scientific Document Group. 2022 ESC Guidelines for the management of patients with ventricular arrhythmias and the prevention of sudden cardiac death. Eur. Heart J..

[33] Armstrong P.W., Pieske B., Anstrom K.J., Ezekowitz J., Hernandez A.F., Butler J., Lam C.S.P., Ponikowski P., Voors A.A., Jia G. (2020). Vericiguat in Patients with Heart Failure and Reduced Ejection Fraction. N. Engl. J. Med..

[34] McMurray J.J., Packer M., Desai A.S., Gong J., Lefkowitz M.P., Rizkala A.R., Rouleau J.L., Shi V.C., Solomon S.D., Swedberg K. (2014). PARADIGM-HF Investigators and Committees. Angiotensin-neprilysin inhibition versus enalapril in heart failure. N. Engl. J. Med..

[35] Teerlink J.R., Diaz R., Felker G.M., McMurray J.J.V., Metra M., Solomon S.D., Adams K.F., Anand I., Arias-Mendoza A., Biering-Sørensen T. (2021). Cardiac Myosin Activation with Omecamtiv Mecarbil in Systolic Heart Failure. N. Engl. J. Med..

[36] Lavalle C., Mariani M.V., Severino P., Palombi M., Trivigno S., D’Amato A., Silvetti G., Pierucci N., Di Lullo L., Chimenti C. (2024). Efficacy of Modern Therapies for Heart Failure with Reduced Ejection Fraction in Specific Population Subgroups: A Systematic Review and Network Meta-Analysis. Cardiorenal Med..

[37] Yancy C.W., Jessup M., Bozkurt B., Butler J., Casey D.E., Drazner M.H., Fonarow G.C., Geraci S.A., Horwich T., Januzzi J.L. (2013). 2013 ACCF/AHA guideline for the management of heart failure: A report of the American College of Cardiology Foundation/American Heart Association Task Force on Practice Guidelines. J. Am. Coll. Cardiol..

[38] Savarese G., Stolfo D., Sinagra G., Lund L.H. (2022). Heart failure with mid-range or mildly reduced ejection fraction. Nat. Rev. Cardiol..

[39] Hamdani N., El-Battrawy I. (2025). Between the Beats: Unraveling Diagnostic, Therapeutic Challenges, and Sex Differences in Heart Failure’s Gray Zone. J. Am. Heart Assoc..

[40] Meijs C., Brugts J.J., Lund L.H., Linssen G.C.M., La Rocca H.B., Dahlström U., Vaartjes I., Koudstaal S., Asselbergs F.W., Savarese G. (2023). Identifying distinct clinical clusters in heart failure with mildly reduced ejection fraction. Int. J. Cardiol..

[41] Solomon S.D., McMurray J.J.V., Vaduganathan M., Claggett B., Jhund P.S., Desai A.S., Henderson A.D., Lam C.S.P., Pitt B., Senni M. (2024). Finerenone in Heart Failure with Mildly Reduced or Preserved Ejection Fraction. N. Engl. J. Med..

[42] Stolfo D., Lund L.H., Sinagra G., Lindberg F., Dahlström U., Rosano G., Savarese G. (2023). Heart failure pharmacological treatments and outcomes in heart failure with mildly reduced ejection fraction. Eur. Heart J. Cardiovasc. Pharmacother..

[43] Kosiborod M.N., Deanfield J., Pratley R., Borlaug B.A., Butler J., Davies M., Emerson S.S., Kahn S.E., Kitzman D.W., Lingvay I. (2024). Semaglutide versus placebo in patients with heart failure and mildly reduced or preserved ejection fraction: A pooled analysis of the SELECT, FLOW, STEP-HFpEF, and STEP-HFpEF DM randomised trials. Lancet.

[44] Anker S.D., Usman M.S., Anker M.S., Butler J., Böhm M., Abraham W.T., Adamo M., Chopra V.K., Cicoira M., Cosentino F. (2023). Patient phenotype profiling in heart failure with preserved ejection fraction to guide therapeutic decision making. A scientific statement of the Heart Failure Association, the European Heart Rhythm Association of the European Society of Cardiology, and the European Society of Hypertension. Eur. J. Heart Fail..

[45] Balestrieri G., Limonta R., Ponti E., Merlo A., Sciatti E., D’Isa S., Gori M., Casu G., Giannattasio C., Senni M. (2024). The Therapy and Management of Heart Failure with Preserved Ejection Fraction: New Insights on Treatment. Card. Fail. Rev..

[46] Uijl A., Savarese G., Vaartjes I., Dahlström U., Brugts J.J., Linssen G.C.M., van Empel V., Brunner-La Rocca H.P., Asselbergs F.W., Lund L.H. (2021). Identification of distinct phenotypic clusters in heart failure with preserved ejection fraction. Eur. J. Heart Fail..

[47] Shahim A., Donal E., Hage C., Oger E., Savarese G., Persson H., Haugen-Löfman I., Ennezat P., Sportouch-Dukhan C., Drouet E. (2024). Rates and predictors of cardiovascular and non-cardiovascular outcomes in heart failure with preserved ejection fraction. ESC Heart Fail..

[48] Capone F., Vettor R., Schiattarella G.G. (2023). Cardiometabolic HFpEF: NASH of the Heart. Circulation.

[49] Tini G., Cannatà A., Canepa M., Masci P.G., Pardini M., Giacca M., Sinagra G., Marchionni N., Del Monte F., Udelson J.E. (2022). Is heart failure with preserved ejection fraction a ‘dementia’ of the heart?. Heart Fail. Rev..

[50] Teramoto K., Ouwerkerk W., Tay W., Tromp J., Katherine Teng T.H., Chandramouli C., Lawson C.A., Huang W., Hung C.L., Chopra V. (2023). Clinical Features of Heart Failure with Normal Ejection Fraction: Insights from the ASIAN-HF Registry. JACC Asia.

[51] Cannata A., McDonagh T.A. (2025). Heart Failure with Preserved Ejection Fraction. N. Engl. J. Med..

[52] Agostoni P., Pluchinotta F.R., Salvioni E., Mapelli M., Galotta A., Bonomi A., Magrì D., Perna E., Paolillo S., Corrà U. (2023). Heart failure patients with improved ejection fraction: Insights from the MECKI score database. Eur. J. Heart Fail..

[53] Kodur N., Tang W.H.W. (2025). Management of Heart Failure with Improved Ejection Fraction: Current Evidence and Controversies. JACC Heart Fail..

[54] Landucci L., Faxén U.L., Benson L., Rosano G.M.C., Dahlström U., Lund L.H., Savarese G. (2025). Characterizing Heart Failure Across the Spectrum of the Preserved Ejection Fraction: Does Heart Failure with Supranormal Ejection Fraction Exist? Data from the Swedish Heart Failure Registry. J. Am. Heart Assoc..

[55] Gao L., Lin Y., Ji M., Wu W., Li H., Qian M., Zhang L., Xie M., Li Y. (2022). Clinical Utility of Three-Dimensional Speckle-Tracking Echocardiography in Heart Failure. J. Clin. Med..

[56] Gosling R.C., Al-Mohammad A. (2022). The Role of Cardiac Imaging in Heart Failure with Reduced Ejection Fraction. Card. Fail. Rev..

[57] Wenzel J.P., Albrecht J.N., Toprak B., Petersen E., Nikorowitsch J., Cavus E., Jahnke C., Riedl K.A., Adam G. (2025). Head-to-head comparison of cardiac magnetic resonance imaging and transthoracic echocardiography in the general population (MATCH). Clin. Res. Cardiol..

[58] Khan M.S., Arshad M.S., Greene S.J., Van Spall H.G.C., Pandey A., Vemulapalli S., Perakslis E., Butler J. (2023). Artificial intelligence and heart failure: A state-of-the-art review. Eur. J. Heart Fail..

[59] Toma M., Mak G.J., Chen V., Hollander Z., Shannon C.P., Lam K.K.Y., Ng R.T., Tebbutt S.J., Wilson-McManus J.E., Ignaszewski A. (2017). Differentiating heart failure phenotypes using sex-specific transcriptomic and proteomic biomarker panels. ESC Heart Fail..

[60] Triposkiadis F., Xanthopoulos A., Drakos S.G., Boudoulas K.D., Briasoulis A., Skoularigis J., Tsioufis K., Boudoulas H., Starling R.C. (2024). Back to the basics: The need for an etiological classification of chronic heart failure. Curr. Probl. Cardiol..

[61] Docherty K.F., Bayes-Genis A., Butler J., Coats A.J.S., Drazner M.H., Joyce E., Lam C.S.P. (2022). The four pillars of HFrEF therapy: Is it time to treat heart failure regardless of ejection fraction?. Eur. Heart J. Suppl..

[62] Forrest I.S., Rocheleau G., Bafna S., Argulian E., Narula J., Natarajan P., Do R. (2022). Genetic and phenotypic profiling of supranormal ejection fraction reveals decreased survival and underdiagnosed heart failure. Eur. J. Heart Fail..

[63] Butler J., Packer M., Filippatos G., Ferreira J.P., Zeller C., Schnee J., Brueckmann M., Pocock S.J., Zannad F., Anker S.D. (2022). Effect of empagliflozin in patients with heart failure across the spectrum of left ventricular ejection fraction. Eur. Heart J..

[64] Inciardi R.M., Riccardi M., Savarese G., Metra M., Vaduganathan M., Solomon S.D. (2025). Tailoring medical therapy for heart failure with preserved ejection fraction. Eur. J. Heart Fail..

[65] Lang R.M., Badano L.P., Mor-Avi V., Afilalo J., Armstrong A., Ernande L., Iachskampf F.A., Foster E., Goldstein S.A., Kuznetsova T. (2015). Recommendations for cardiac chamber quantification by echocardiography in adults: An update from the American Society of Echocardiography and the European Association of Cardiovascular Imaging. J. Am. Soc. Echocardiogr..

[66] Savarese G., Schiattarella G.G., Lindberg F., Anker M.S., Bayes-Genis A., Bäck M., Braunschweig F., Bucciarelli-Ducci C., Butler J., Cannata A. Heart failure and obesity: Translational approaches and therapeutic perspectives. A scientific statement of the Heart Failure Association of the ESC. Eur. J. Heart Fail..

